# Applications and challenges of rhodopsin-based optogenetics in biomedicine

**DOI:** 10.3389/fnins.2022.966772

**Published:** 2022-09-23

**Authors:** Hanci Zhang, Hui Fang, Deqiang Liu, Yiming Zhang, Joseph Adu-Amankwaah, Jinxiang Yuan, Rubin Tan, Jianping Zhu

**Affiliations:** ^1^College of Life Sciences, Shandong Normal University, Jinan, China; ^2^Department of Physiology, Basic Medical School, Xuzhou Medical University, Xuzhou, China; ^3^Collaborative Innovation Center for Birth Defect Research and Transformation of Shandong Province, Jining Medical University, Jining, China; ^4^Lin He’s Academician Workstation of New Medicine and Clinical Translation, Jining Medical University, Jining, China

**Keywords:** optogenetics, microbial rhodopsin, Alzheimer’s disease, Parkinson’s disease, cardiac optogenetics, optical cochlear implant

## Abstract

Optogenetics is an emerging bioengineering technology that has been rapidly developed in recent years by cross-integrating optics, genetic engineering, electrophysiology, software control, and other disciplines. Since the first demonstration of the millisecond neuromodulation ability of the channelrhodopsin-2 (ChR2), the application of optogenetic technology in basic life science research has been rapidly progressed, especially in neurobiology, which has driven the development of the discipline. As the optogenetic tool protein, microbial rhodopsins have been continuously explored, modified, and optimized, with many variants becoming available, with structural characteristics and functions that are highly diversified. Their applicability has been broadened, encouraging more researchers and clinicians to utilize optogenetics technology in research. In this review, we summarize the species and variant types of the most important class of tool proteins in optogenetic techniques, the microbial rhodopsins, and review the current applications of optogenetics based on rhodopsin qualitative light in biology and other fields. We also review the challenges facing this technology, to ultimately provide an in-depth technical reference to support the application of optogenetics in translational and clinical research.

## Introduction

Optogenetics is a technique that integrates the fields of optics, genetic engineering, electrophysiology, and software control to regulate specific activity in cells with the help of various light-responsive proteins (Kim et al., 2017). Compared with chemical molecules and electrical signals, optical signals used in optogenetics have higher temporal and spatial resolution and cause lower damage to cells. As such, they have been widely used in basic research such as neurobiology, cell biology, and molecular biology (Kolesov et al., 2021).

Light-responsive proteins are a class of proteins commonly found in nature that can perform specific functions in response to stimulation by specific light wavelengths. For instance, they can mediate ion transmembrane transport, gene expression, and cell signal transduction (Govorunova et al., 2017a; Duan et al., 2022). Through genetic engineering optimization of optogenetics, a series of optogenetic tool proteins have been successfully developed. Microbial rhodopsins are one of the most widely used. At the beginning of this century, channelrhodopsins (ChRs) were first evaluated and functionally identified, opening the door to optogenetics research (Nagel et al., 2002, 2003). Boyden et al. used channelrhodopsins-2 (ChR2) to successfully modulate the activity state of neurons in the isolated hippocampus, thereby ushering in the Optogenetics 1.0 era of neurobiological research (Boyden et al., 2005; Nagel et al., 2005; Bi et al., 2006). With the construction of additional subtypes and variants of light-responsive proteins, the scope of application of optogenetics has been greatly expanded, including in a wider range of organisms. For instance, the introduction of *Gt*ACR1 into the guard cells of tobacco by Huang et al. was used to show that the continuous stimulation of green light caused depolarization of the plasma membrane, which led to voltage-gated K^+^ channels opening, guard cell swelling, and stomatal closure; this is a prerequisite technical condition for analyzing the effect of patchy stomatal conductance on water use efficiency. This crosses the limits of plant and animal species and applies optogenetic techniques to the field of plant physiology, enabling artificial control of stomatal closure and broadening the application areas of the technology. Optogenetics technology provides a time-saving and labor-saving technical tool for the study of long-duration plant physiological phenomena (Huang et al., 2021). In a more complex biological reaction process, such as the LicV system described by Liu *et al.*, which can be an effective and tunable optogenetic control of transcriptional and genomic locus markers is achieved (Liu et al., 2022). In subsequent studies, the sources of optogenetic tool proteins have become more diverse, such as UV-B, cryptochrome (CRY), and light-oxygen-voltage (LOV) domains derived from plant photoreceptor proteins. The implicated biological reaction processes span from the regulation of gene expression to cell signal transduction, and from macroscopic neurobiological research to microscopic molecular and cell biology research. These works have facilitated and promoted the technical research of optogenetics in the Optogenetics 2.0 era (Tan et al., 2022). The utility of optogenetics 2.0 has moved beyond ion transport control to the exploration of multiple sources of instrumental proteins, photoreceptor modification, and the use of different types of light to control more complex biochemical reactions in cells. Organisms that have been studied using optogenetics have broadened from animals to plants and microorganisms. Therefore, in addition to neuroscience, optogenetics 2.0 provides a powerful problem-solving approach for disciplines such as plant science, cell biology, and molecular biology.

Attempts have been made to merge optogenetic technologies with clinical medical research, boosting its translation into clinical research applications and even into clinical practice. To date, such research has mainly focused on cardiac pacing and heart rate regulation, visual acuity recovery, optical cochlear implant (oCls), and other fields. For example, optogenetic treatment was used to effect the partial recovery of visual function in a patient with retinitis pigmentosa, providing the first report of its use in a neurodegenerative context (Sahel et al., 2021). In this paper, we review the current variants of microbial rhodopsins, provide a comprehensive description and systematic evaluation of the potential applications of rhodopsin-based optogenetics in the biomedical field, and provide an outlook of the opportunities and challenges facing its future development. We believe that rhodopsin-based optogenetics will not only play an important leading role in basic research but also have a strong potential for biomedical and clinical applications. However, many issues need to be considered such as the safety of viral vectors, trafficking of the protein, targeting light to the protein, and the efficiency of the process. Prevailing over these challenges may allow optogenetics to leap from biomedical to clinical translational applications.

## Microbial rhodopsins

**FIGURE 1:**
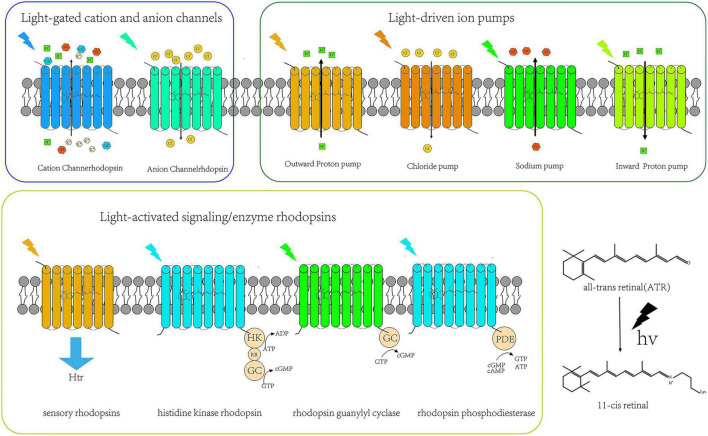
Types of microbial rhodopsins.

From the invention of optogenetics to the present decade, the family size of microbial rhodopsins (opsin + ovalently-bound retinal = rhodopsin) has been expanding, and its variants have been continuously enriched. Based on its structure and functional characteristics, rhodopsins can be divided into three categories: light-driven ion pump, light-gated ion channel, and light-activated signaling/enzyme rhodopsins. The emergence of new microbal rhodopsin mutants not only opens up the range of optogenetic applications in basic research but also provides a powerful weapon for using optogenetics technology to explore the pathogenic mechanisms, diagnosis, and treatment of various neurological diseases, such as neurodegenerative diseases. The classifications of the microbial rhodopsin family of proteins are shown in Figure 1.

### Light-driven ion pumps

As early as 1971, researchers discovered the first microbial Bacteriorhodopsin (BR) in *Halobacterium salinarum*. This is a light-driven hydrogen ion pump that mediates hydrogen ion transmembrane outflow and participates in photosynthetic phosphorylation (Oesterhelt and Stoeckenius, 1971; Oesterhelt and Stoeckenius, 1973; Danon and Stoeckenius, 1974). BR provided several milestones for structural biology as the first detailed and thoroughly studied microbial rhodopsin, the first seven-transmembrane protein with a resolved molecular configuration, and the first membrane protein with a determined amino acid sequence (Khorana et al., 1979). Unfortunately, at the time, its unique transmembrane ion transport mode did not attract the attention of neurobiologists. It was only when channelrhodopsin-2 (ChR2) was discovered and started to be exploited in neurobiology that BR was gradually adopted by neuroscientists (Boyden et al., 2005). Subsequently, more hydrogen ion pumps homologous to BR were discovered, such as Arch (Chow et al., 2010) and Mac (Husson et al., 2012). Arch, a hydrogen ion pump from *Halorubrum sodomense*, is usually used to suppress neuronal activity, but it is more noteworthy as a genetically encoded voltage indicators (GEVIs) for the real-time imaging of brain action discharges (Kralj et al., 2011). Mac is a fungal hydrogen ion pump. Husson et al. (2012) found that Mac can control the behavior of *caenorhabditis elegans* by inhibiting neuronal activity. There are also many other exciting potential photogenetic tool proteins in fungi. For instance, the recently discovered fungal ApOps series of bacteriorhodopsin are also photo-controlled ion pump bacteriorhodopsins. For example, this protein can be localized on the plasma membrane of specific organelles, such as lysosomes, and green light can be used to change their internal and external acid-base environments to study the biological consequences (Panzer et al., 2021).

The second microbial rhodopsin to be discovered was the Cl^–^ ion pump-Halorhodopsin (HR) (Matsuno-Yagi and Mukohata, 1977). In the HR family, the most mature application is *Np*HR, which was found in *Natronomonas pharaonis*. *Np*HR mediates the transmembrane influx of Cl^–^ by ∼590 nm light thereby silencing neurons; this has mainly been used to explore the neural circuit architecture of animals during critical behavioral activities (Lanyi, 1986; Zhang et al., 2007; Chen et al., 2021). However, its disadvantage is that the generated photocurrents are low, which limits its effectiveness in inhibiting neuronal activity. As a result, the behavioral phenotype remains relatively unaffected thus limiting its utility in research. Subsequently, various variants have been developed based on wild-type *Np*HR, such as e*Np*HR (Gradinaru et al., 2008), e*Np*HR 2.0 (Gradinaru et al., 2009) and e*Np*HR 3.0. The modified and optimized e*Np*HR 3.0 protein is more highly enriched and localized on the cell membrane, rather than in the cytoplasm, which allows it to produce greater photocurrents while having little effect on the intracellular environment. This variant has been used as a neurosilencing tool in optogenetics (Gradinaru et al., 2010).

From the 1970s and over the following 40 years, no new light-driven pumps for aprotic cations were been reported, except for the chloride and hydrogen ion pump. In 2013, Inoue K et al. reported that they isolated a light-driven Na^+^ ion pump-KR2 from *Krokinobacter eikastu* (also known as DeNaR), and that KR2 can pump sodium ions out of cells to achieve neuronal silencing (Inoue et al., 2013; Hososhima et al., 2021). Later, Shevchenko V et al. described an internal flow H^+^ pump rhodopsin (Inoue et al., 2016; Shevchenko et al., 2017). Studies have shown that the largest advantage of proton pumps over chloride pumps is that they produce higher photocurrent rates and have less interference with γ−aminobutyric acid (GABA) energy interneurons (Chow et al., 2010). With the continuous discovery of the ion pump type of rhodopsin, the types of optogenetics tool proteins are becoming greatly enriched, making it possible for optogenetics to be applied in wider scientific research fields and more disciplines. The summary of light-driven ion pump rhodopsins are shown in Table 1.

**TABLE 1:** Widely used naturally occurring microbial rhodopsins.

Name	Absorption peak (nm)	Source	Descriptions	Year	References
**Light-driven ion pumps**					
BR	560	*Halobacterium salinarum*	The first microbial rhodopsin identified as a light-driven hydrogen ion pump.	1971	Nagel et al., 2005
Arch	566	*Halorubrum sodomense*	Also known as Archaerhodopsin-3 (AR3), used as genetically encoded voltage indicators (GEVIs).	2010	Chow et al., 2010
Mac	550	*Leptosphaeria maculans*	A light-gated H^+^ outflow pump derived from fungi.	2012	Husson et al., 2012
*Hs*HR	588	*Halobacterium salinarum*	The second found microbial rhodopsin. it is a Cl^–^ internal flow pump.	1977	Matsuno-Yagi and Mukohata, 1977
*Np*HR	590	*Natronomonas pharaonic*	The most widely used Cl^–^ internal flow pump.	1986	Lanyi, 1986
KR2	524	*Krokinobacter eikastus*	A ligh-driven Na^+^ outflow pump.	2013	Inoue et al., 2013
*Po*XeR	567	*Parvularcula Oceani*	A light-driven H^+^ internal flow pump.	2016	Inoue et al., 2016
**Light-gated cation and anion channels**					
*Cr*ChR1	495	*Chlamydomonas reinhardtii*	ChR1, the first cationic channel protein, discovered from *Chlamydomonas rheinissima*, has high permeability to H^+^.	2002	Nagel et al., 2002
*Cr*ChR2	470	*Chlamydomonas reinhardtii*	ChR2, non-selective cation channel protein, is the most widely used and variant of rhodopsin.	2003	Nagel et al., 2003
VChR1	589	*Volvox carteri*	The first evaluated red-shift cation channel microbial rhodopsin.	2008	Zhang et al., 2008
ChRmine	520	*Rhodomonas lens*	Red shift, high photosensitivity, fast kinetics.	2019	Marshel et al., 2019
VirChR1	507	*Organic Lake phycodnaviru*	No Ca^2+^ permeability.	2020	Zabelskii et al., 2020
*Gt*CCR4	540	*Guillardia theta*	High Na^+^ permeability, low H^+^/Ca^2+^ permeability, high photosensitivity, rapid kinetics.	2020	Hososhima et al., 2020
Chrimson	590	*Chlamydomonas noctigama*	CnChR1, it has a red-shift excitation spectrum.	2014	Klapoetke et al., 2014
Chronos	480	*Stigeoclonium helveticum*	ShChR, high photosensitivity and fast kinetics.	2014	Klapoetke et al., 2014
*Gt*ACR1	515	*Guillardia theta*	The first evaluated light-controlled anion channel rhodopsin.	2015	Govorunova et al., 2015
*Gt*ACR2	470	*Guillardia theta*	Excitation spectrum blue shift	2015	Govorunova et al., 2015
ZipACR	520	*Proteomonas sulcata*	It shows a larger photocurrent amplitude and a faster conductivity cycle.	2017	Govorunova et al., 2017b
RapACR	520	*Rhodomonas salina*	Rapid dynamics	2020	Govorunova et al., 2020
*Hc*KCR1	540	*Hyphochytrium catenoides*	It is a natural light-gated potassium channel, which is high photosensitivity and fast kinetics	2022	Govorunova et al., 2022
**Light-activated signaling/enzyme rhodopsins**					
HKR	380 and 490	*Chlamydomonas*	Rh-UV state absorbs ultraviolet light (380nm) into Rh-Bl state, and Rh-Bl can absorb blue light (490nm) into Rh-UV	2012	Luck et al., 2012
Rh-GC	520	*Blastocladiella emersonii* and *Catenaria anguillulae et al*.	The ability to produce cGMP by cycination.	2014	Avelar et al., 2014
Rh-PDE	492	*Salpingoeca rosetta*	The ability to produce cGMP and cAMP by cycination.	2017	Yoshida et al., 2017

### Light-gated cation and anion channels

Channelrhodopsin-2 (ChR2) is derived from *Chlamydomonas reinhardtii*; this genus is the earliest and most widely used microbial rhodopsin (Boyden et al., 2005; Kim et al., 2017). ChR2 is a light-controlled non-selective cation channel protein that mediates the inward flow of Na^+^, K^+^, H^+^, Ca^2+^, and other cations across the membrane under blue light (420 nm) stimulation, resulting in the depolarization and activation of neurons. It is widely used in the analysis and functional characterization of neuronal circuits. In addition, other ion channel rhodopsins are naturally present in nature. The unique redshift properties of VChR1 from *Volvox carteri*, as reported by Zhang et al., provided an important reference for other genetically engineered rhodopsin variants (Zhang et al., 2008); Marshel et al. obtained ChRmine with high photosensitivity and high electrical conductivity through biogenic analysis and gene mining, which can be activated by light to a depth of 7cm in the cerebral cortex, which suggests that it has broad applicability (Marshel et al., 2019; Chen et al., 2020); VirChR1, a viral rhodopsin discovered by Zabelskii et al. (2020) in Antarctica, blocks Ca^2+^ penetration, effectively solving the problem of neurotransmitter release from the presynaptic membrane caused by Ca^2+^ penetration and avoiding the adverse effects on downstream neuronal activities. Other wild-type light-gated ion channel microbial rhodopsins are presented in Table 1.

Most excitingly, A new type of engineed rhodopsin developed by targeted mutations or chimeric modifications of the ChR protein family is particularly well characterized for various scenarios of optogenetic applications. Based on their excellent properties, the variants can be roughly classified into three categories: kinetic opsins, red-shifted opsins, and step-function opsins (SFO) (Kim et al., 2017).

Kinetic opsins are ChR2 variants with high-frequency excitation and rapid inactivation properties, including ChETA (Gunaydin et al., 2010), CHIEF (Lin et al., 2009) and ET/TC (Lin et al., 2013). These variants are excited by high-frequency excitation light and can better simulate the excitation state of neurons under natural conditions, which is of great significance for studying neural encoding. Alternatively, step-function opsins (SFO) include stabilized step-function opsin (SSFO) (Yizhar et al., 2011) and step-waveform inhibitory ChR (SwiChR) (Berndt et al., 2015); this is a class of opsins that can maintain the state of neurons for a long time by brief light excitation and their function can be decreased gradually by a certain order of magnitude, thus explaining the name step-function opsin. For example, they can be used for prolonged, chronic neuromodulation of specific neurons or neuronal circuits.

Due to the low tissue penetration of ChR2 stimulated by blue light, it cannot meet the demand of deep brain projection. Zhang et al. (2008) developed the first red-shifted optic protein C1V1 using an assembly chimera. However, the remote projection performance of C1V1 is inadequate and is not yet able to transmit the signal to the projection end of neural circuits. Subsequently Lin et al. (2013) developed another red-shifted opsin-ReaChR. Although ReaChR can deliver a sufficient amount of opsin to the end of a neural circuit, it is poorly compatible with mammals and can only induce a weak photocurrent with poor fidelity. Later, Rajasethupathy et al. combined the above-mentioned research strategies and developed variants with high photocurrent, redshiftable, and high teleprojection abilities using the system of target mutation and chimera assembly-bReaChES (Rajasethupathy et al., 2015). In addition, the red-shifted property of the excitation light in redshift proteins allows the combined use of redshift and blueshift proteins, and genetically encoded voltage indicators (GECIs), for multiple photorecording and simultaneous acquisition of neural circuits.

The aforementioned ion pump rhodopsins for neuronal silencing can only transport a single ion during the photocycle induced by a single photon, thus showing low ion transport efficiency. Therefore, a more desirable neurosilencing tool would be a natural anion-conducting channelrhodopsin like the cation-conducting ChR2. In 2014, Deisseroth and Hegemann independently designed and successfully obtained the anionic channel rhodopsins: iC1V1 (Berndt et al., 2014) and ChloC (Wietek et al., 2014). Deisseroth obtained iC1V1 *via* a targeted mutation at 9 positions (T98S/E129S/E140S V156K/E162S H173R/V281K/T285N/N297Q) based on the molecular structure information of the C1V1. Hegemann obtained ChloC by introducing two mutation sites (E90R/T159C) into ChR2. However, the original iC1V1 still transported H^+^, and the optimized, and successfully obtained iC + + and iChloC have higher selectivity and conductivity to Cl^–^ in two variants while solving the efficiency problem (Berndt et al., 2015). Meanwhile, Spudich isolated naturally occurring ACRs from *Guillardia Theta*, including *Gt*ACR1 and *Gt*ACR2. These ACRs overcame the shortcomings of conventional ion pump transport efficiency, such that ACR can transfer 10^4^–10^5^ ions per second and exhibit a 10^2^–10^4^-fold higher photosensitivity than the inhibitory ion pump rhodopsin. In recent years, an increasing number of ACRs have been obtained through genome mining and engineering, such as ZipACR (Govorunova et al., 2017b), RapACR (Govorunova et al., 2018), and RubyACR (Govorunova et al., 2020). Recently, Govorunova *et al.* reported that the long-sought kalium channelrhodopsins (KCRs) from *Hyphochytrium catenoides*, *Hc*KCR1 and *Hc*KCR2, show tunable inhibition of mouse cortical neurons with millisecond precision, providing a promising optogenetic tool for the study and treatment of Parkinson’s disease, epilepsy, and other disorders (Govorunova et al., 2022).

Along with engineering modifications of rhodopsins, novel variants with many favorable characteristics have been developed. For instance, rhodopsins that can simultaneously activate and inhibit the same neuron in a single experiment, such as the eNPAC2.0 (NpHR-TS-p2A-hChR2(H134R)-EYFP) designed by Carus-Cadavieco et al. (2017). The experimental requirement of yellow light (586/20 nm) for inhibition and blue light (475/28 nm) for activation helped to elucidate the neural loop mechanisms involved in foraging behavior. Unfortunately, the results were not completely satisfactory. The reliability of blue light neuronal has not yet reached 100% (5 Hz: shows 84.0 ± 11.0% and 20 Hz shows 65.0 ± 12.0) (Carus-Cadavieco et al., 2017; Gao et al., 2021). Vierock et al. (2021) further optimized the blue-light-activated *Gt*ACR2 and red-light-activated Chrimson chimerism to obtain BiPOLES, which effectively solved the above problems and realized that the inhibitory anionic and excitatory cationic currents were observed maximum photocurrent density in different excitation light peak. The effect was validated in Drosophila melanogaster, mice, and ferrets, thereby satisfying the need for bidirectional and simultaneous control of neuronal activity in a single experiment (Vierock et al., 2021). The above examples demonstrate that engineered modified bicolor rhodopsins can meet the demand for rapid switching of activating/inhibiting neuronal activity and have great potential for biomedical applications. More variant types and characteristics are detailed in Table 2.

**TABLE 2:** Widely used engineered variants.

Name	Template	Engineering method	Mutations	Characteristics	Time	References
ChR2 H134R	ChR2	Point mutations	H134R	The first widely used variant type of ChR2.	2005	Nagel et al., 2005
ET/TC	ChR2	Point mutations	E123T/T159C	High frequency and fast kinetics.	2010	Berndt et al., 2011
ChR2 T159C	ChR2	Point mutations	T159C	High photosensitivity, fast kinetics.	2011	Berndt et al., 2011
ChETA	ChR2	Point mutations	E123T	Fast kinetics.	2010	Gunaydin et al., 2010
ChIEF	ChEF	Point mutations	I170V	Fast kinetics.	2009	Lin et al., 2009
C1V1	TM1-3 source CrChR1 and TM3-7 source VChR1	Chimera	−	Excitation spectrum redshift.	2008	Zhang et al., 2008
ReaChR	N-terminal is derived from ChR1,TM1-5 and TM-7 from VChR1, and TM6 from VChR2	Chimeras and point mutations	L171I	Excitation spectrum redshift.	2013	Lin et al., 2013
bReaChES	N-terminal is derived from ChR2 and ChR1,TM1-5 and TM7 from VChR1, and TM6 from VChR2	Chimeras and site-directed mutations	E123S/L132I	Excitation spectrum redshift, strong long-distance transport.	2015	Rajasethupathy et al., 2015
CatCh	ChR2	Point mutations	L132C	High Ca^2+^ permeability, high photosensitivity and fast kinetics.	2011	Kleinlogel et al., 2011
ChrimsonR	Chrimson	Point mutations	K176R	Rapid desensitization.	2014	Klapoetke et al., 2014
Vf-Chrimson	Chrimson	Point mutations	K176R/Y261F/S267M	Fast kinetics.	2018	Mager et al., 2018
SSFO	ChR2	Point mutations	C128S/D156A	Slow desensitization.	2011	Yizhar et al., 2011
SwiChR	iC1C2	Point mutations	C167A or C167T	Slow desensitization.	2016	Berndt et al., 2015
SOUL	SSFO	Point mutations	T159C	High photosensitivity.	2020	Gong et al., 2020
iC1V1	C1C2	Point mutations	T98S/E129S/E140S /V156K/E162S/H173R /V281K/T285N/N297Q	There’s some proton leakage.	2014	Berndt et al., 2014
ChloC	*Cr*ChR2	Point mutations	E90R/T159C	There’s some proton leakage_°_	2014	Wietek et al., 2014
iC + +	iC1C2	Point mutations	T98S/E122N/E129Q/E140S /V156R/E162S /V281R/T285N/N297Q/E312S	No proton leakage, high Cl^–^ permeability.	2016	Berndt et al., 2015
iChloC	ChloC	Point mutations	E83Q/E90R/E101S /D156N/T159C	No proton leakage, high Cl^–^ permeability.	2015	Govorunova et al., 2015
BiPOLES	*Gt*ACR2 and Chrimson	Chimera	−	Double color stimulate.	2021	Vierock et al., 2021

### Light-activated signaling/enzyme rhodopsins

The first class of signaling/enzyme rhodopsins was discovered in 1982 in *Halobacterium. Salinarum*; these included sensory rhodopsin I (SRI) (Spudich and Spudich, 1982) and sensory rhodopsin II (SRII, also known as phoborhodopsin, pR) (Bogomolni and Spudich, 1982). Sensory rhodosins (SRs) are embedded in the cell membrane in seven transmembrane alpha helices. When excited, SRs convert the light signal to the cognate transducer halobacterial transducer proteins (Htrs), and form 2:2 complexes with their cognate transducer proteins (halobacterial transducer protein for SRI (HtrI) and halobacterial transducer protein for SRII (HtrII)), which causes a downstream cascade reaction leading to flagellar motility and participation in phototropism. Other signaling/enzyme rhodopsins were subsequently identified. Unlike the previous microbial rhdopsins, the subsequently identified signaling/enzyme rhodopsins are eight-transmembrane (8-TM) proteins, with the C- and N-terminal ends of the protein located on the cytoplasmic side. All the identified signaling/enzyme rhodopsins exist as dimers, including histidine kinase rhodopsin (HKR) (Luck et al., 2012), guanylate cyclase (Rh-GC) (Avelar et al., 2014), and rhodopsin phosphodiesterase (Rh-PDE) (Yoshida et al., 2017). It has been demonstrated that light-controlled signaling/enzyme rhodopsins can be utilized to solve basic science problems. For example, Henss et al. expressed Rh-GC in *Cryptobacterium hidrophilum* and used this to control the locomotion speed of nematodes *via* light activation (Henss et al., 2022).

## Biomedical applicability of rhodopsins

With the advent of optogenetics, there has been an uptake of application in basic biomedical medicine research, such as in the exploration of the mechanism of neurodegenerative diseases. Simultaneously, clinical applications of optogenetics have been explored, including in areas such as vision restoration and optical cochlear implants (oCIs).

### Alzheimer’s disease

Alzheimer’s disease (AD) is a neurodegenerative disease that typically occurs in middle-aged and elderly people. With an increasing aging population, the number of AD patients is rising every year (Scheltens et al., 2021). According to current epidemiological data, the global prevalence of AD will increase three folds by 2050. Current research on the pathogenesis of AD has generated a number of pathophysiological hypotheses that include amyloid-beta (Aβ) toxicity (Selkoe and Hardy, 2016), tau protein hyperphosphorylation (Liang et al., 2022), and synaptic dysfunction (Skaper et al., 2017). Optogenetics provides a novel approach to the study of AD pathogenesis and its clinical treatment. Zhang et al. (2020) used ChR2 to drive GABA neurons and found that this could attenuate neuroinflammation and amyloid β protein levels in the hippocampal CA1 region of APP/PS1 mice and help to restore memory. Guillaume Etter et al. expressed ChETA in MSPV cells and found that activation of parvalbumin interneurons at a 40 Hz frequency restored the normal hippocampal slow-wave amplitude and phase-amplitude coupling in the J20 amyloid β AD mouse model (Etter et al., 2019). Roy et al. reported that the reduction in dendritic ridge density of DG neurons could be reversed by optogenetic techniques in animals with early AD, and that activation of DG engram cells using optogenetic techniques could also restore situational memory (Roy et al., 2016). The aforementioned animal experiments suggest that optogenetic techniques may provide a potential avenue for the treatment of AD. At present, because of the multiplicity of neuronal degenerative lesions in the AD brain, current treatments generally target the entire brain. In addition, most viral vectors need to be embedded in the host cell genome to achieve stable expression, yet there are no effective means to achieve the simultaneous integration of viral sequences within the host genome of the whole brain. As such, current optogenetic techniques are not yet matured enough to be exploited to target the whole brain.

### Parkinson’s disease

Parkinson’s disease is another clinically common neurodegenerative disorder that begins with the degenerative death of dopaminergic neurons in the substantia nigra of the midbrain, eventually leading to abnormalities in the glutamatergic excitatory pathways in the cerebral cortex, limbic system to the basal ganglia, and motor areas of the brainstem spinal cord (Bloem et al., 2021). Clinical symptoms are mainly motor abnormalities, such as slow and increasing motor dysfunction in individuals, and non-motor clinical symptoms such as cognitive impairment, depression, and pain. The neuropathological markers of PD include the presence of Lewy bodies and Lewy neurites (neuronal inclusions immunopositive for the protein α-synuclein); similar pathological changes have been demonstrated in skin, blood, colon and other tissues (Shahmoradian et al., 2019). Optogenetic stimulation of glutamatergic neurons in the cuneiform nucleus of the midbrain motor area, which in turn activates glutamatergic excitatory pathways extending to the motor areas of the brainstem spinal cord, has been shown to effectively improve dyskinesia in a mouse PD model (Fougère et al., 2021). Deep brain stimulation (DBS) is a common and effective treatment method utilized in clinical practice; combining DBS with optogenetics can be used to explore the neural mechanisms that underlie symptomatic improvement in response to DBS (Gittis and Yttri, 2018). We have reason to believe that, as optogenetic technology improves, it will prove to be critical to revealing the pathogenic mechanisms of Parkinson’s disease and in aiding patient treatment.

Astrocytes are an important component of the tripartite synapse structure; they receive neurotransmitter signals through cell surface receptors and regulate synaptic activity *via* gliotransmitters. In recent years, research on neurodegenerative diseases has begun to focus on glial cells. Xie et al. (2019) reported that activation of signaling pathways related to synaptic activity in astrocytes by optogenetic techniques affects the release of gliotransmitters, which can modulate the communication network between astrocytes and neurons. Other studies have focused on microglia, which are another important neuroglial cell that plays a major role in phagocytosis and immunomodulation. Microglial activation can be accompanied by the inward flow of chloride ions which further initiates their phagocytic activity. The introduction of chloride ions could be achieved by *Np*HR, and we speculate that this may offer a potential therapeutic application if microglia are specifically activated in PD lesioned brain regions, which in turn initiates phagocytosis of α-synuclein (Averaimo et al., 2010).

### Cardiac optogenetics

Similar to neurons, cardiomyocytes are also excitable cells. Thus, optogenetics can be used to regulate their physiological activity. Optogenetics have been exploited in the study of the heart for around 12 years (Entcheva and Kay, 2020). In 2014, Bingen et al. transfected CatCh into neonatal rat cardiomyocytes as a strategy to defibrillate the heart through cell activation, thereby suggesting an optogenetic therapeutic strategy for patients with atrial fibrillation (Bingen et al., 2014). Such an approach would address some of the limitations of electric defibrillators, such as reducing the risk of myocardial tissue damage and pain. A recent paper utilizing computational simulation pointed out that *Gt*ACR1 may be used to defibrillate human hearts (Ochs et al., 2021). Majumder et al. (2020) developed a theoretical ion channel called the Biologically Integrated Cardiac Defibrillator (BioICD), whose activation can lead to a rapid and repeated restoration of normal rhythm for arrhythmia in the human atrium and ventricle (Majumder et al., 2020). It is believed that this theoretical simulation may soon be developed. Unlike the nervous system however, cardiac optogenetics lacks well-characterized tissue-specific promoters.

### Ophthalmic optogenetics

Ophthalmic optogenetics is the earliest biomedical application of optogenetics. Bi et al. (2006) pioneered the expression of ChR2-encapsulated AAV vectors in retinal ganglion cells (RGCs), demonstrating the potential of optogenetics in visual recovery (Bi et al., 2006). Several variants of ChR2 introduced earlier have shown promising ophthalmic applications. For example Sengupta et al. (2016) used ReaChR carried by the AAV vector and injected into the rd1 mouse model of blindness and orange light with an intensity lower than the human safety threshold to restore retinal, cortical, and behavioral levels of light-response. Chuong et al. (2014) showed that Jaws-encapsulated AAV vectors achieved light responses from isolated ON- and OFF-retinal ganglion cells in transgenic mice after 600 nm light stimulation. Recently, ChrimsonR was successfully transfected using AAV into a patient’s retinal ganglion cells, resulting in a partial recovery of the degenerated retina. This is the first successful optogenetics case in neurodegenerative disease (Sahel et al., 2021). In addition, four companies have promoted ophthalmic optogenetics in clinical trials: (1) GenSight Biologics, GS030(Paris, France), (2) Allergan, RST-001 (Dublin, Ireland), (3) Bionic Sight, BS01(New York, NY, USA), and (4) Nanoscope Therapeutics, vMCO-010 (Bedford, TX, USA) (Prosseda et al., 2022). Although optogenetics has advanced our ability to effect the recovery of visual acuity, there are marked differences between optogenetic vision and natural vision. One is that photoreceptor cells can detect the stimulation intensity of a single photon, and there is a significant difference in sensitivity. The other is that optogenetic vision is not yet involved in the formation of color perception. If different light wavelength optogenetic tool proteins are expressed in specific cell types, color or color-like perception may emerge. However, it is believed that optogenetics will continue to play a strong role in ophthalmic research and clinical practice.

### Optical cochlear implants

Electronic cochlear implants (eCIs) are currently the primary clinical alternative to the organ of Corti for restoring hearing function. When hearing loss or hearing impairment is severe, eCIs bypass the sensory hair cells to directly convert sound information into a digital signal, which is reprocessed into a radio frequency (RF) signal that is embedded in the patient’s skin with an internal receiver and stimulator. The RF is decoded and the spiral ganglion neurons (SGNs) are directly stimulated along the electrodes to produce hearing (Zeng et al., 2008). The drawbacks of eCIs include a low frequency resolution due to the wide distribution of electrode contacts and current diffusion, etc., which makes them less effective in noisy environments. Optical cochlear implants (oCIs) modulate the sound signal into an optical signal with the assistance of an acousto-optic modulator, which directly replaces electrical stimulation with optical stimulation. This process improves the frequency resolution of artificial sound encoding due to a lower spatial spread of neural excitation. Currently, Keppeler et al. have established the expression system of CatCh by AAV in rodents and experimentally validated LED-based piggyback oCIs. Usually, LED-based cochlear implants carry 10-15 LED exciters (emitters), while μLED-based oCIs can increase the number of emitters to 144 emitters of 60 × 60 lm, which has been established by utilizing 16 microscale thin-film light-emitting diodes (μLEDs) for oCIs (Klein et al., 2018; Dieter et al., 2020; Keppeler et al., 2020). A recent study showed that the Chrimson variants f-Chrimson and vf-Chrimson also have a potential for optical cochlear implant applications (Bali et al., 2021). At present, the development of oCIs is still in its formative stages, and researchers are working hard to find better matching conditions for rhodopsins and to develop oCIs with higher frequency resolution. It is believed that this work will facilitate the design of next generation oCIs and facilitate their clinical translatability.

## Challenges of optogenetics in the biomedical sphere

Optogenetics has empowered basic research scientists and clinicians to precisely manipulate cellular activity. Its application to basic research and therapeutics has attracted numerous research teams. The ultimate goal is the widespread safe and effective use of optogenetics in the human body. One step toward this goal would involve solving the problem of light delivery, such as through the development of wireless optogenetics. In this section we discuss some of the most pressing and core issues in the therapeutic translatability of optogenetics.

### Selection and delivery of viral vectors

As is the case with other gene therapies, optogenetics requires a vehicle to deliver its proteins to specific cell types; viruses are the ideal transporters. Adeno-associated viruses (AAV) are one of the most widely used viral tools in basic research and clinical trials. As a non-pathogenic member of the parvoviridae family, AAV is composed of a single-stranded DNA genome encapsidated in a 23-28 nm, T = 1, non-invariant capsid (El Andari and Grimm, 2020). Three serotypes of AAV have been licensed for clinical use, including Glybera (AAV1), Luxturna (AAV2), and Zolgensma (AAV9) (Domenger and Grimm, 2019). These viruses are used as opsin transporters for the following reasons: (1) they are non-encapsulated viruses, which are more stable and less likely to break down under laboratory conditions; (2) they are wild-type viruses without virulent potential; although this increases their safety, immunogenicity must be considered. Because AAV is prevalent in humans, most patients carry antibodies to AAV, so they may respond to endogenous AAV vectors through pre-existing immunity (PEI). Therefore, there is a need to develop isolates or serotypes that do not trigger such responses or that only display mild immunogenicity. Another strategy is to modify the gene sequence of the antigenic part of the AAV sequence so that its antigen cannot bind antibodies in the circulatory system to minimize the immune response induced by AAV, thus increasing the efficiency and possibility of targeting cells; (3) they are efficient opsin transporters, including in non-human primates. Their application in non-human primate studies provides sufficient experimental data and theoretical support for the clinical application of optogenetics, as non-human primates have a high similarity with humans in terms of anatomical structure and genetic information; (4) they are free from host DNA, stable by satellite form, and cannot integrate into the host genome. A recent study, in which AAV2-CAG-ChrimsonR:tdTomato was injected into the vitreous humor of macaques demonstrated that optogenetics-mediated RGCs activity persisted for at least 12 months after 12 weeks of transfection (McGregor et al., 2022).

In addition, the precise delivery of the viral vector to the target cells is also problematic. Currently, mainly stereotactic techniques are used, but they carry the risk of infection and may not allow all desirable regions to be accurately targeted. Alternative techniques include ventricular, spinal, and vascular injection modalities, but these methods have their own problems. Because AAVs are hepatophilic, most are injected into the organism and become distributed to the liver, which may produce hepatotoxicity and reduce their targetability. To obtain AAV serotypes that can better target the CNS, PHP.B (Deverman et al., 2016) and PHP.S (Challis et al., 2019) were designed. Recently, scientists have shown organ-specific targeting of adeno-associated virus (AAV) capsids after intravenous delivery, which we achieved by employing a Cre-transgenic-based screening platform and sequential engineering of AAV-PHP.eB between the surface-exposed AA452 and AA460 of VP3. This variant exhibits good CNS targeting and low hepatotropism and has been validated in *Callithrix Jacchus* (Goertsen et al., 2021).

### Light delivery

Light delivery is another major challenge for the clinical utilization of optogenetics. In traditional basic optogenetics research, stereotactic positioning followed by fiber optic implantation is usually adopted. There are certain drawbacks to using this method: the surgery is difficult to precisely perform and leaves the animals at risk of infection. In addition, the tissue damage caused by the fiber optic implants is irreversible and behavioral activities are easily restricted by a combination of the surgery and attendant equipment. Moreover, the heat generated by the light can cause phototoxicity (Owen et al., 2019). Previous studies have shown that activation of the blue light-activated stGtACR2 opsin, expressed in the hippocampus of rats, increased the local peak brain temperature, thereby significantly decreasing the population spike (PS) amplitudes and latencies of Eps, thus confounding the central experimental methods (Acharya et al., 2021). The emergence of wireless optogenetics has solved the problem of wired fiber implants. Researchers have developed an improved optical transmission system that transmits light through a wireless headset and uses small light-emitting diodes (LEDs) to activate the rhodopsin mass, eliminating the need for optical fibers and allowing for wireless charging (Kwon et al., 2015; Montgomery et al., 2015). Recently, a less invasive wireless optogenetic technique has been developed using light with wavelengths shorter than the visible spectrum, such as ultraviolet or X-ray with wavelengths < 450 nm or higher wavelengths, such as infrared light with wavelengths > 650 nm; this inorganic scintillator has been used for the bidirectional modulation of neurons (Miyazaki et al., 2019; Matsubara et al., 2021).Therefore, wireless optogenetics is expected to help advance the uptake of optogenetic-based clinical treatments.

It is worth noting that most of the excitation wavelengths of the current optogenetic tool protein - microbial rhodopsins - involve visible light (400nm-700nm). However, if blue light optic violet matter is used in very deep target brain regions, wireless optogenetics may appear airy and ineffective. Although the use of redshift protein has solved part of the problem, gaps remain in the clinical application of optogenetics (Chen, 2019). Therefore, both traditional and wireless technical means are limited by the microbial rhodopsins itself. Upconversion nanoparticles (UCNPs) can absorb near-infrared light and emit visible light from ultraviolet to near-red light. Compared with traditional optical fiber implantation, UCNP-mediated optogenetics has the advantages of less tissue damage, wireless transmission, and negligible toxicity, More importantly, it reduces the consideration of microbial rhodopsins properties. One interesting report demonstrated a UCNP with a photoreceptor for anchoring retinal photoreceptors and sending signals to the brain upon near-red light stimulation, allowing mammals to see infrared light (Ma et al., 2019); other applications of UCNPs in biomedicine have also been summarized (Mahata et al., 2021).

## Conclusion

Rhodopsin-based optogenetics have been widely used in various fields of life science research. Although their development in the field of clinical medicine is still in its early stages, their great potential in this area is well recognized. For the sake of brevity, in this review, we focused on the application of optogenetics in several specific fields of medicine, but we recognize that their utility will become more widespread in the future. In the field of autism, optogenetics has been used to activate the glutamatergic BF-VTA pathway, leading to an immediate shift from sleep to awakening in mice. This showed that excessive excitation of the glutamatergic BF-VTA pathway is associated with clinical disorders characterized by excessive defensive behaviors (Cai et al., 2022). In regenerative medicine, a recent article reported that optogenetic tools improve the differentiation of primary murine myotubes (Chapotte-Baldacci et al., 2022). In addition, optogenetics plays an important role in revealing depression-like neural circuits (Biselli et al., 2019). Of course, many problems need to be overcome. The selection and transmission of viral vectors and optical transmission are some of the major core issues. In addition, immunogenicity, cell specificity, and other challenges stemming from the use of viral vectors need to be urgently solved. To solve these problems, experiments must be conducted on non-human primates. At present, an Open Resource for Non-human Primate Optogenetics has been established (Tremblay et al., 2020), which will provide important technical support for the experimentation of optogenetic technology in non-human primates and promote its clinical translation.

It is clearly that optogenetic techniques have unique applications and prospects for development, but more importantly their combination with other techniques will provide strategies and technical means to solve many problems. One of the promising research directions is closed-loop experiments based on optogenetics and optical imaging techniques, but an ideal algorithm for the accurate identification of active neurons is lacking. Sheng et al. constructed analysis software called Online Real-time activity and offline Cross-session Analysis (ORCA), which performs image alignment, neuron segmentation, and activity extraction at over 100 frames per second; this is fast enough to support real-time neuron detection and readout activity, providing a solution for full optical closed-loop control of neuronal activity (Sheng et al., 2022). This will provide software support for real-time feedback control of neurons based on neuronal activity, with far-reaching promise in addressing neurological disorders such as epilepsy. We believe that optogenetics will play an important role in advancing basic research and that it will ultimately greatly benefit patients.

## Author contributions

JZ, RT, and JY: conceptualization. HZ, HF, DL, and YZ: methodology. HZ and JA-A: formal analysis. JZ, HZ, RT, and JY: resources. JZ, HZ, HF, DL, and YZ: writing-original draft. JA-A and RT: revising and editing. All authors: writing-review and editing, investigation.
